# Ultra-Low-Cost 3D Bioprinting: Modification and Application of an Off-the-Shelf Desktop 3D-Printer for Biofabrication

**DOI:** 10.3389/fbioe.2019.00184

**Published:** 2019-07-31

**Authors:** Melanie Kahl, Markus Gertig, Phillipp Hoyer, Oliver Friedrich, Daniel F. Gilbert

**Affiliations:** Institute of Medical Biotechnology, Friedrich-Alexander-University Erlangen-Nürnberg, Erlangen, Germany

**Keywords:** ultra-low-cost bioprinting technology, alginate and alginate/gelatin-based hydrogel, HEK293^YFPI152L^ cells, rapid prototyping, 3D desktop printing

## Abstract

3D bioprinting has become a versatile and powerful method in tissue engineering and regenerative medicine and is increasingly adapted by other disciplines due to its tremendous potential beyond its typical applications. However, commercially available 3D bioprinting systems are typically expensive circumventing the broad implementation, including laboratories in low-resource settings. To address the limitations of conventional and commercially available technology, we developed a 3D bioprinter by modification of an off-the-shelf 3D desktop printer, that can be installed within a single day, is of handy size to fit into a standard laminar flow hood, customizable, ultra-low cost and thus, affordable to a broad range of research labs, or educational institutions. We evaluate accuracy and reproducibility of printing results using alginate and alginate/gelatin-hydrogels and demonstrate its potential for biomedical use by printing of various two-and three-dimensional cell-free and mammalian cell-laden objects using recombinant HEK^YFP^ cells, stably expressing yellow fluorescent protein (YFP) as a model system and high-content imaging. We further provide a parts list and 3D design files in STL and STEP format for reconstructing the device. A time-lapse video of the custom-built device during operation is available at https://vimeo.com/274482794.

## Introduction

Three-dimensional (3D) bioprinting has become a versatile and powerful method for generating a variety of biological constructs, including bone or extracellular matrix scaffolds, endo- or epithelial, tumor, or muscle tissue as well as organoids (Tan et al., 2014; Zhao et al., 2014; Fahmy et al., 2016; Carter et al., 2017; Mir and Nakamura, 2017; Hong et al., 2018). Due to its tremendous potential for a large variety of fields of research, including animal-free *in vitro* drug or toxicity screening, 3D bioprinting is increasingly being aspirated by disciplines besides its typical domains of tissue engineering and regenerative medicine. However, 3D bioprinting requires specific infrastructure that is mostly expensive thus, slowing down its integration with other disciplines. The costs of conventional and commercially available 3D bioprinting technology range between tens of thousands to several hundreds of thousands euros, strongly limiting its applicability to a small number of specialized laboratories. Thus, despite being a cutting-edge technology that is relevant to a broad community, it is not readily applicable for research in low-resource settings or even for educational purposes, e.g., in primary or secondary schools or universities. Also, common 3D bioprinters are typically complicated to use and tie up highly skilled staff for application and maintenance. Despite this fact, many systems are restricted to generating very simple, e.g., two-dimensional scaffolds only. As hard- and software is mostly closed source, modification, or customization for generation of more complex structures is prevented. Furthermore, commercially available devices are often bulky and immobile and allow operation under non- or semi-sterile conditions only, which—in the worst case—can cause failure of a planned experiment. Therefore, despite its vast potential, the methodology is obviously still under-adopted and much less exploited than it could be, as if the required technology was available to a broader range of laboratories.

Despite the fact that there are a number of examples on bioprinting technologies of reduced/low-cost of several hundred euros (Wang et al., 2015; Goldstein et al., 2016; Reid et al., 2016; McElheny et al., 2017; Roehm and Madihally, 2017), a 3D bioprinter, that is straightforward to use, portable, customizable, and available within a price range of ~€ 150, i.e., that is ultra-low-priced, has not been reported yet.

The emergence of rapid prototyping technologies, including open source microcontroller architecture, low priced electronics, mechanics, and robotics parts as well as consumer grade additive manufacturing techniques, such as desktop 3D printing, is currently revolutionizing the character of scientific lab automation infrastructure throughout all research disciplines of life sciences (Chhaya et al., 2015; Walzik et al., 2015; Schneidereit et al., 2016; Delalat et al., 2017; Shukla et al., 2017; Schmitt et al., 2019). Rapid prototyping technology—also aspired by the so-called *Maker* movement, a culture of do-it-yourself (DIY) product generation (Landrain et al., 2013; Seyfried et al., 2014)—makes it possible to quickly and easily engineer user-friendly devices of reduced complexity and of ultra-low-cost.

To address the limitations of conventional and commercially available technology described above, we aimed to develop a 3D bioprinter based on a *Makers* approach, that can be installed within a single day, is of handy size to fit into a standard laminar flow hood, customizable, ultra-low cost and thus, affordable to a broad range of research labs, and educational institutions. To this end, we aimed to modify an off-the-shelf Desktop 3D Printer to carry a 1-milliliter sterile and disposable syringe for printing of hydrogels. In order to demonstrate the applicability of our device to biomedical research for two- and three-dimensional printing, we intended to conduct a case study with different types of cell-free and mammalian cell-laden alginate-based hydrogels. For evaluation of cellular viability in cell-laden bioconstructs over extended time periods, we aimed to analyze cell proliferation using fluorescence microscopy. We decided to use alginate-based hydrogel because of its biocompatibility and gelation properties making it useful for bioprinting (Wang et al., 2003; Abbah et al., 2008; Hunt et al., 2009; Ab-Rahim et al., 2013; Hunt and Grover, 2013; Sarker et al., 2014; Tabriz et al., 2015). For our case study, we aimed to use recombinant HEK^YFP^ cells, stably expressing yellow fluorescent protein (YFPI152L) as a model system. The cell line allows microscopic evaluation and quantitative analysis of cellular proliferation and viability in fluo-micrographs obtained from high-content imaging of cell-laden hydrogels and thus, analysis of biofabricated constructs generated using the in house-built ultra-low-cost 3D bioprinter (Schneidereit et al., 2016; Kuenzel et al., 2017; Milanos et al., 2018). Finally, we aimed to provide detailed information to re-building the device, including a comprehensive parts list as well as 3D design files in STEP and STL format.

## Results

**Figure 1:**
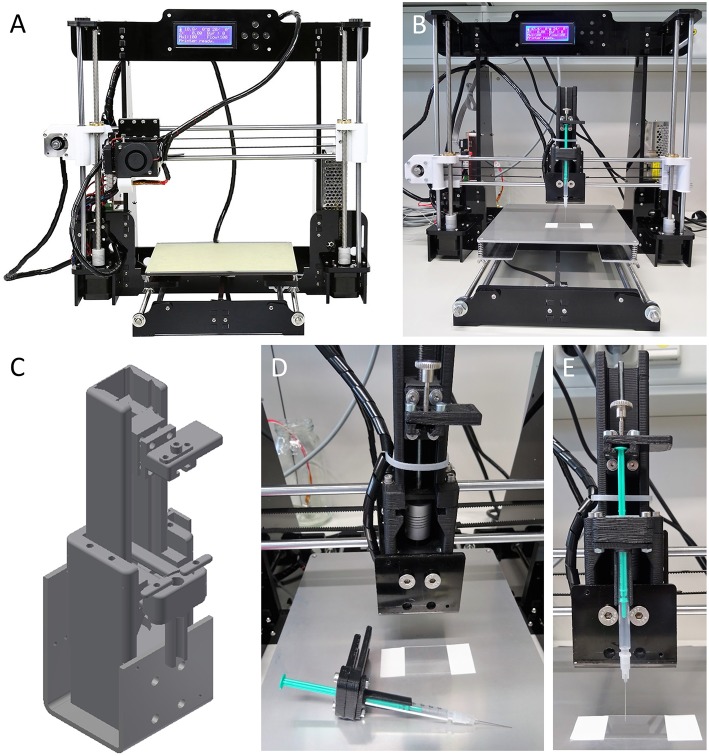
In house-built 3D bioprinter. The printer is ultra-low-cost (~€ 150), small (510 × 400 × 415 mm) and of light weight (8.5 kg). **(A)** Off-the-shelf bioprinter (Anet A8 Desktop 3D Printer Prusa i3 DIY Kit) in its original configuration and prior to modification. The printer is shipped as a kit and can be assembled within a few hours with support of a large variety of video tutorials and websites available in many different languages on the internet. **(B)** Ultra-low-cost 3D bioprinter after modification of the printer shown in **(A)**. Most parts required for modification of the device can be fabricated using the off-the-shelf desktop printer prior to its modification using, e.g., acrylonitrile butadiene styrene. Only few components, including a threaded rod, a spring coupling and standard screws, and nuts are need to be purchased but may also be fabricated from 3D printing. **(C)** Three-dimensional representation of a syringe unit suitable to carrying and operating an Injekt-F disposable 1-milliliter syringe for printing of cell-laden hydrogel. An animated drawing showing the individual components of the syringe unit is available at https://vimeo.com/274482812. 3D design files in STL and STEP format for reconstruction of the ultra-low-cost 3D bioprinter are provided in the Supplementary Material. **(D,E)** Enlarged visualizations of the 3D printed syringe unit without **(D)** and with **(E)** mounted disposable 1-ml syringe.

We have developed an ultra-low-cost 3D bioprinter by modifying an off-the-shelf desktop 3D-printer that can easily be replicated within a few hours. Most of the components required for modification of the consumer grade desktop 3D printer are either part of the original 3D printer, or can be can be fabricated with the original device prior to its conversion. The printer is ultra-low-cost (€ ~150) small (510 × 400 × 415 mm) and light-weight (8.5 kg) and thus, portable and applicable within a standard laminar flow hood. To demonstrate the applicability of the resource-effective printer to biomedical research, we applied it with recombinant HEK^YFP^ cells as a model system. We further provide a parts list and 3D design files for reconstructing the device. The combination of its ultra-low-costs, availability, portability, applicability, and customizability is worldwide unique and unmet by other 3D bioprinting systems. The device is shown in Figure 1B. A detailed parts list is included in the Supplementary Material. A time-lapse video of the custom-built device during operation is available at https://vimeo.com/274482794.

### Conversion of a Desktop 3D Printer Into an Ultra-Low-Cost 3D Bioprinter

As a starting point toward developing an ultra-low-cost 3D bioprinter, we aimed to select a basic 3D desktop printer model that is resource-efficient and of high precision and at the same time, broadly supported by a vast user community with respect to application and trouble shooting. Having the aforementioned aspects in mind, we decided to purchase an Anet A8 Desktop 3D Printer Prusa i3 DIY Kit (Anet, see Figure 1A) and assembled it within a few hours based on the large variety of video tutorials and websites being available in many different languages on the internet.

In a next step, for conversion of the original 3D printer into a 3D desktop bioprinter, we aimed to manufacture a 3D printable syringe unit for handling of cell-laden hydrogels or bioinks. To this end, we designed a syringe unit suitable of carrying and operating an Injekt^®^-F disposable 1-milliliter syringe (Braun) using CAD (computer-assisted-design) software (Autodesk Inventor). We decided to implement this type of syringe into our system for a number of reasons. First, the syringe is sterile and fabricated for medical use thus, suitable to application with *in vitro* cell cultures. Second, the plunger of the syringe is equipped with a conical spine to reduce dead volume, facilitating applicability, and shortening the experimental lead-time prior to biofabrication. Third, the tip of the syringe consists of a versatile luer lock fitting for connection with various luer lock compatible needle types which may be purpose-selected based on the cell line or bioink to be used and/or the construct to be fabricated. Fourth, the overall dimensions of the syringe allow integration with the pre-selected desktop 3D printer and fifth, the syringe's volume is suitable for printing two-dimensional monolayers of extended area or large three-dimensional constructs, e.g., cube-shaped, with a side length of 10 mm.

To keep the conversion of the original 3D printer into a 3D bioprinter simple, straightforward and time- as well as cost-effective, we intended to use as many existing components of the original set-up as possible for the syringe unit, including stepper motor, and connectors. A schematic representation of the 3D model of the unit is shown in Figure 1C. The unit is made up of a total of 48 components, including screws and nuts, of which in our case 15 were 3D printed from black acrylonitrile butadiene styrene (see Methods for details), but that could also be fabricated using any other manufacturing technique from different material. Printing of the parts took ~8 h. Besides the 3D printed parts, the unit is made-up of a threaded rod (: 4 mm, M4) attached to the stepper motor of the original 3D printer via spring coupling (www.gearbest.com). A detailed parts list is included in Table S1 in the Supplementary Material. Assembly of the syringe unit takes ~30 min. An animated drawing showing the individual components of the syringe unit is available at https://vimeo.com/274482812. The fully assembled modified ultra-low-cost 3D bioprinter with installed syringe unit and mounted 1-ml syringe is shown in Figure 1B. Enlarged visualizations of the syringe unit with and without mounted 1-ml syringe are shown in Figures 1D,E, respectively.

### 3D Printing Characteristics

The usability of a 3D bioprinter for biofabrication is limited by a variety of parameters, including its overall traveling range and speed, the extrusion rate, its spatial resolution, i.e., the smallest traveling distance of the system in X-, Y-, and Z-direction and its accuracy, i.e., the difference between the actual printing result and the expected result based on a provided CAD model as quantified by a measurement technique (Conrad et al., 2000).

The traveling range of the 3D printer in its original configuration is 220 × 220 × 240 mm (X-Y-Z axis). However, in the modified system, the overall travel range is reduced to ~100 × 100 × 240 mm (X-Y-Z axis) as the syringe unit is displaced toward the center of the printing bed with respect to the printing origin in the original 3D printer. Based on information provided by the manufacturer, the spatial resolution, i.e., the positioning accuracy in the X-Y-and Z-direction, is 12 and 4 μm, respectively.

**Figure 2:**
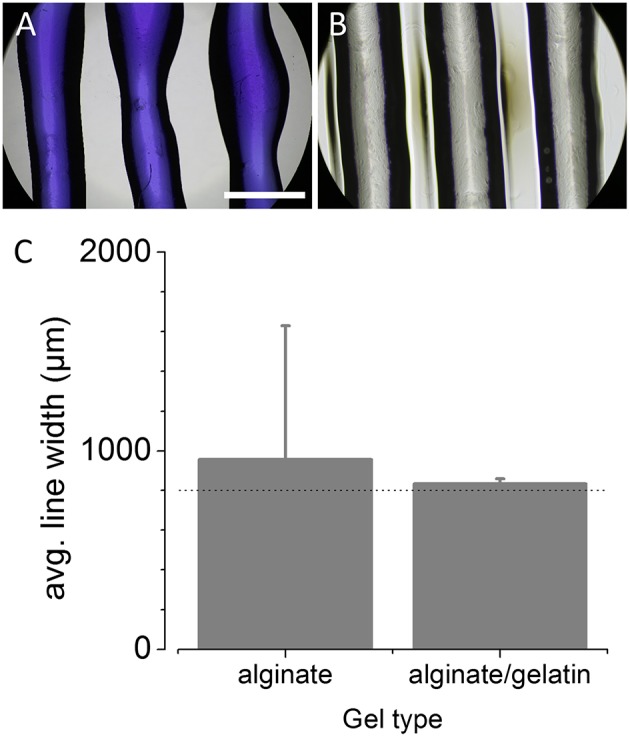
Evaluation of printing parameters after conversion of the off-the-shelf printer into a 3D bioprinter. **(A,B)** Representative microscopic images of bromophenol blue-5% alginate hydrogel **(A)** and 5% alginate/5% gelatin hydrogel **(B)** lines, printed onto a glass coverslip and captured using a 4x objective. Scale bar: 1 mm. **(C)** Histogram of the width (in μm) of the printed lines (mean ± SD) for evaluation of the printing accuracy and repeatability for 5% alginate and a mixture of alginate and gelatin. The dotted line represents the target line width of 800 μm. These data demonstrate the suitability of the ultra-low-cost 3D bioprinter to printing of different hydrogels and indicate moderate to high accuracy and reproducibility.

Besides the aforementioned positioning accuracy, the printing accuracy also strongly depends on the diameter of the printing nozzle or needle, the feed or extrusion rate of the bioink as well as the overall printing speed. The larger the needle-diameter, the smaller the resolution and the less accurate the printing result. The higher the extrusion rate at a given needle diameter, the less accurate the printing result and the higher mechanical stress for the biological probe. As for biofabrication in general, the viability of the printed biological probe is superior to the printing accuracy, and the printing accuracy in turn is superior to the printing speed, the speed always needs to be adjusted to allow a high level of printing accuracy at maximal viability of the printed probe. In order to avoid mechanical stress on the printed cells and at the same time operate the printer with high accuracy, we used needles with a relatively large diameter (400 μm for 5% alginate and 800 μm for alginate/gelatin) as compared to the positioning accuracy of the printer. In this configuration and based on simple CAD line models, we optimized both, extrusion rate and printing speed for printing 5% alginate hydrogel and a mixture of 5% alginate/5% gelatin, bioinks commonly used in biofabrication (see Methods for details). The optimum settings were selected by qualitatively comparing the printing result with the expected outcome, i.e., the CAD models, and were found to be a printing speed of 100 mm/s and an extrusion rate of 550–600 steps/mm for 5% alginate hydrogel as well as a printing speed of 3 mm/s and an extrusion rate of 300 steps/mm for the alginate/gelatin mixture. Using those values as our default settings for subsequent experiments, we assessed the accuracy of the printer as described in the Methods section. In brief, lines were repeatedly printed onto a rectangular glass coverslip using the same syringe/needle. To increase the contrast for subsequent analysis, 5% alginate hydrogel was stained with bromophenol blue to increase the contrast for subsequent analysis. Due to comparably higher contrast of the alginate/gelatin mixture, this hydrogel was left unstained for analysis of printing characteristics. The printed constructs were then imaged in transmission light using a microscope with 4x objective, and the dimensions of the printing result were quantitatively analyzed using image analysis software (see Methods for details). Figures 2A,B show representative example images for 5% alginate and the alginate/gelatin mixture, respectively. The histogram in Figure 2C shows the mean width (± SD) of the printed lines calculated from a total of 13 individual constructs (955 ± 673 μm, *N* = 39 lines, left) for 5% alginate as well from three constructs for the mixture of alginate and gelatin (834 ± 23 μm, *N* = 9 lines, right), indicating large variation of the printing result using 5% alginate and the 400 μm needle and much smaller variation with alginate/gelatin hydrogel and the 800 μm needle.

These data demonstrate the suitability of the ultra-low-cost 3D bioprinter to printing of various hydrogels with moderate to high accuracy and reproducibility with respect to the dimensions of the extruded material.

### Biofabrication Case Studies With the Ultra-Low-Cost Printer and HEK293^YFP^ Cells

In order to assess whether the ultra-low-cost 3D bioprinter is suitable for printing of mammalian cell-laden hydrogels, we aimed to fabricate differently structured objects and to culture the probes for several days as well as to analyse cellular proliferation by microscopic evaluation and image analysis. To this end, we added 10^6^ HEK293^YFP^ cells into 1 ml 5% alginate-hydrogel or 5% alginate/5% galatin-hydrogel as described in the Methods section. HEK293^YFP^ cells stably express yellow fluorescent protein (YFP). As printed objects only emit YFP fluorescence light in presence of viable cells, this approach allows direct correlation of the fluorescence signal with the number of viable cells as well as cell proliferation in printed bioconstructs.

### Printing of 2D Bioconstructs

**Figure 3:**
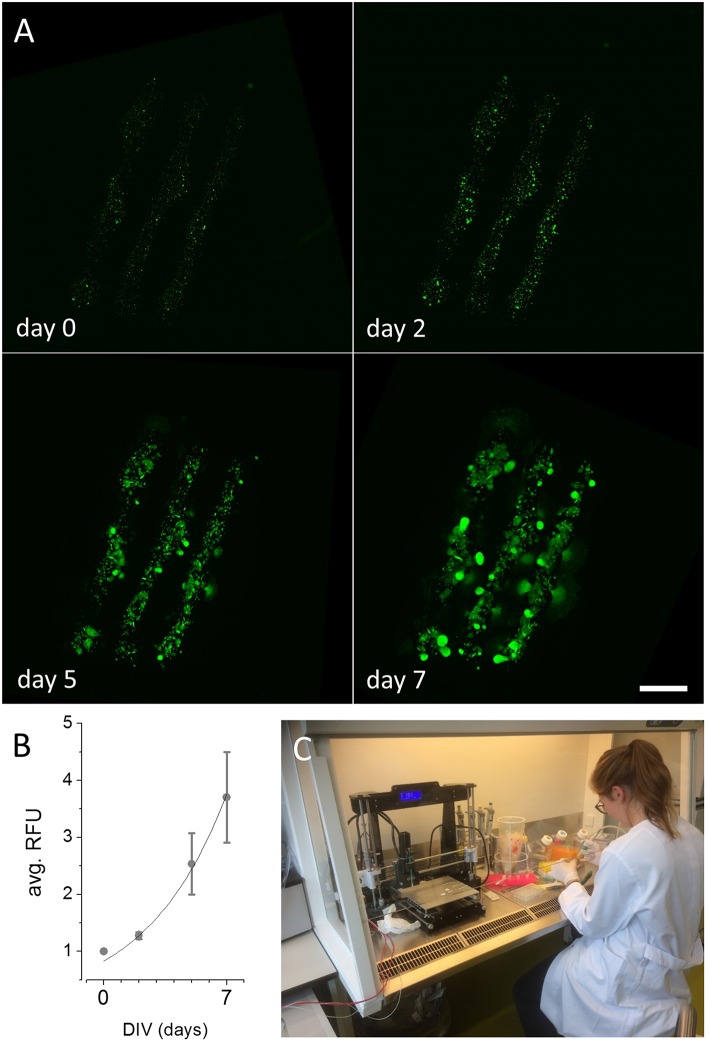
Maturation of 2D printed recombinant HEK293^YFP^ cell-laden alginate-hydrogel using the ultra-low-cost system. **(A)** Reconstructed and registered fluo-micrographs of the printed structure shown in Figure 2A after 0, 2, 5, and 7 days *in vitro*, respectively, indicating cellular proliferation and viability within the bioconstruct. These data demonstrate the suitability of the in house-built system for ultra-low-cost bioprinting. Scale bar: 2 mm. **(B)** Time-course of average relative fluorescence intensity (RFU, mean ± SD) during long-term culture as indicator of cellular proliferation of HEK293^YFP^ cells. The dotted line represents an exponential fit indicating exponential growth of the cells within the hydrogel and high cellular viability. **(C)** 3D bioprinting in our lab during operation. Due to its light-weight and small dimensions, the device can be operated within a standard laminar flow hood under sterile conditions. Written informed consent was obtained from the individual for the publication of this image.

Figure 3A and Figure S1B show reconstructed images of printed 2D structures after 0, 2, 5, and 7 days *in vitro*, respectively. Image sequences of the fluo-micrographs for the models depicted in Figure 2A and Figure S1A are available at https://vimeo.com/275028609 and https://vimeo.com/275028500, respectively. For quantification of cellular proliferation in the printed cell-laden hydrogel shown in Figure 3A during the culture period, we measured the mean pixel intensity within each of the printed lines for every analyzed time point using image analysis software. A time-course of fluorescence intensity (mean ± SD, *N* = 3) as indicator of cellular viability and proliferation in cell-laden hydrogel is shown in Figure 3B. An exponential fit to the plotted data (solid black line in Figure 3B, *R*^2^ = 0.99) indicates exponential growth of the recombinant kidney-derived cells in the biofabricated three-dimensional construct and thus, indicates high viability of the printed cells. An image of the device during experimental use with cells within a laminar flow hood is shown in Figure 3C. These results clearly demonstrate that the ultra-low-cost 3D bioprinter is suitable for two-dimensional printing of mammalian cell-laden alginate-based hydrogel.

### Printing of 3D Bioconstructs

**Figure 4:**
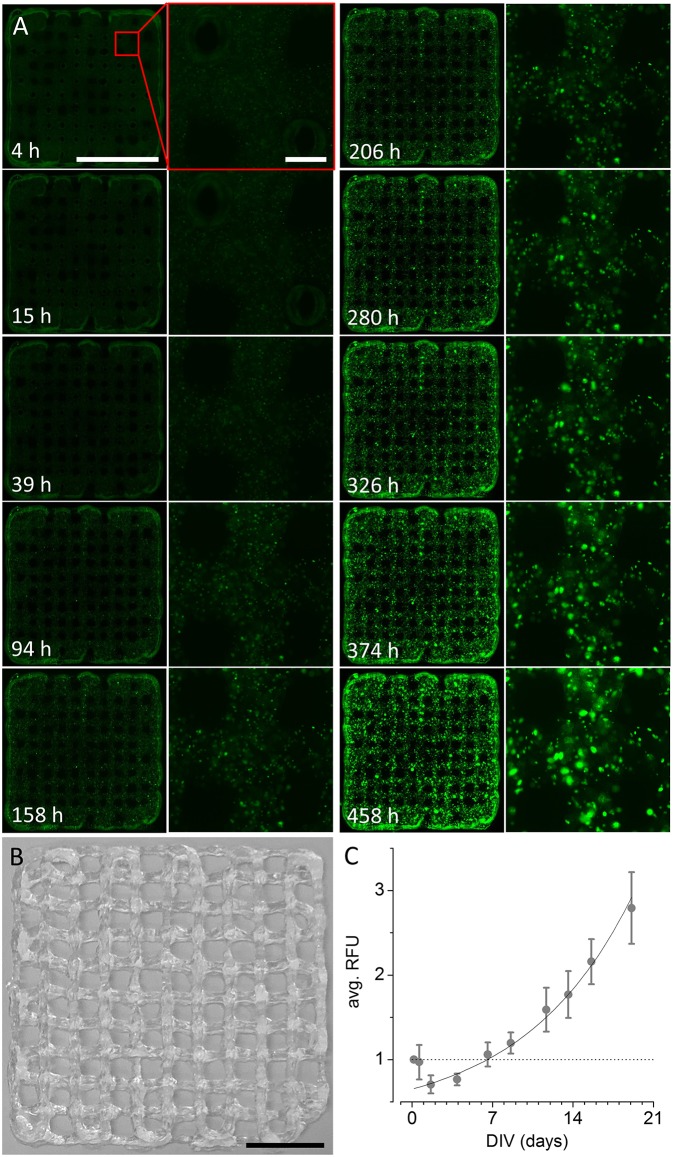
Long-term culture of 3D printed recombinant HEK293^YFP^ cell-laden alginate/gelatin-hydrogel using the modified 3D printer. **(A)** Reconstructed and registered fluo-micrographs (left) and magnified regions (right, indicated by red rectangle) of a 3D printed grid as shown in **(B)** 4, 15, 39, 94, 158, 206, 280, 326, 374, and 458 h *in vitro*, respectively. Scale bars: 10 mm (left), 500 μm (right). **(B)** Photograph of 3D printed alginate/gelatin-hydrogel grid, constructed using the ultra-low-cost system. Scale bar: 10 mm. **(C)** Time-course of the normalized and averaged relative fluorescence intensity (RFU, mean ± SD) during long-term culture as indicator of cellular proliferation and viability. The dotted line represents the fluorescence signal at experiment start and indicates that the cellular viability is altered immediately after printing. The solid line represents an exponential fit indicating fast recovery of cellular viability followed by exponential growth of the cells within the hydrogel. These data clearly demonstrate the suitability of the in house-built system for ultra-low-cost bioprinting.

**Figure 5:**
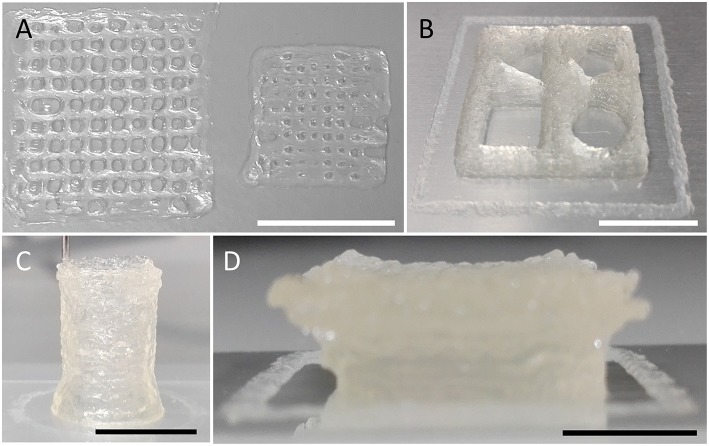
3D printing of various geometries and shapes indicating the versatility of the ultra-low-cost bioprinter. **(A)** Images of differently sized grids constructed by printing of orthogonally orientated lines with varying spacing of 1 (left) and 0.5 mm (right). The smaller object indicates the limitations of the used needle/hydrogel combination for printing of lines separated by ~500 μm. Due to the fluidic nature of the hydrogel, closely neighboring lines merge upon printing, resulting in a disordered grid structure. **(B)** Square-shaped object of 20 mm edge length and 2 mm height, enclosing four different geometrical shapes (triangle, top-left; pentagon, top-right; rectangle, bottom-left; circle, bottom-right). **(C)** Tube (hollow cylinder) of 9 mm diameter and 14 mm height. **(D)** Overhanging structure (Half-cut and upside-down pyramid with concave top) indicating the stability of printed objects. Scale bars: 10 mm.

To evaluate whether the ultra-low-cost printer is also suitable for printing of three-dimensional, multi-layered objects, we fabricated differently sized, rectangular grids by printing orthogonally orientated lines of alginate/gelatin-hydrogel with varying spacing of 2, 1, and 0.5 mm. An example of a grid with a line spacing of 2 mm is shown in Figure 4B. Example images of smaller grids with line spacing of 1 (left) and 0.5 mm (right) are shown in Figure 5A. The smallest object on the right side in Figure 5A indicates the limitations of the used hydrogel/needle configuration for printing lines separated by only 500 μm. Due to the fluidity (i.e., low viscosity) of the hydrogel, parts of the neighboring lines merge upon printing, resulting in a disordered grid structure. Grids of larger line spacing however, showed a more homogeneous structure and were thus used for long-term culture and analysis of cell proliferation and viability. Figure 4A shows reconstructed and registered fluo-micrographs (left columns) and magnified regions (right columns) of a 3D printed grid with 2 mm line spacing after 4, 15, 39, 94, 158, 206, 280, 326, 374, and 458 h *in vitro*, respectively. Figure 4C shows the time-course of the normalized and averaged relative fluorescence intensity (RFU, mean ± SD) during long-term culture as indicator of cellular proliferation and viability. The dotted line represents the fluorescence signal at experiment start and indicates that the cellular viability is altered immediately after printing. The solid line represents an exponential fit (*R*^2^ = 0.94) indicating fast recovery of cellular viability followed by exponential growth of the cells within the hydrogel. These data clearly demonstrate the suitability of the in house-built system for ultra-low-cost bioprinting.

### Printing of Three-Dimensional Objects of Large Dimensions

Three-dimensional constructs to be generated using the ultra-low-cost system may be much larger than shown in Figures 2–4, respectively. To demonstrate that the device used in the present study also allows fabrication of larger 3D objects, we printed constructs of different size and shape as shown in Figure 5 and Figure S2 using cell-free alginate/gelatin-hydrogel. These data indicate the versatility and applicability of the ultra-low-cost system for generation of three-dimensional objects of various geometries.

## Materials and Methods

### Design and Fabrication of the Syringe Unit for Modification of an Off-the-Shelf 3D Printer

Components of the syringe unit were designed using computer-assisted design (CAD) software (Autodesk Inventor 2013, Autodesk, Inc., USA) and were 3D printed from black acrylonitrile butadiene styrene (ABS, MakerBot Industries, USA) using a MakerBot Replicator 3D printer (MakerBot Industries, USA). We decided to fabricate the components of the syringe unit with another 3D printer than the device we aimed to modify, as this facilitated design optimization and circumvented the need for multiple assembly-disassembly-cycles, accelerating the prototyping procedure.

### Cell Culture

Recombinant HEK293^YFP^ cells, stably expressing YFP were used in bioprinting experiments. The fluorescent protein allows microscopic evaluation and quantitative analysis of cellular proliferation and viability in fluo-micrographs obtained from high-content imaging of cell-laden hydrogels and thus, analysis of biofabricated constructs generated using the in house-built ultra-low-cost 3D bioprinter. The HEK293^YFP^ cell line was generated as previously described in Walzik et al. (2015) and was cultured in Dulbecco's modified Eagle's medium (DMEM, Invitrogen) supplemented with 10% fetal calf serum and penicillin (100 U/ml)/streptomycin (100 mg/ml) (Sigma-Aldrich, USA) at 37°C, 5% CO_2_ in a humidified incubator according to standard procedures. Cells were passaged every 2–3 days and used in experiments when ~80–90% confluent.

### Preparation of Hydrogels

3D printing experiments were conducted with alginate-based hydrogels because this type of bioink is simple to produce and the viscosity of the gel can be varied via the alginate concentration or by addition of additives such as gelatin. 5% (w/v) sodium alginate (Sigma Aldrich, USA) was dissolved in pre-warmed (37°C) phosphate buffered saline (PBS, Life Technologies, USA) and stirred at 50°C for 2–3 h. For enhancing the contrast of the gel during evaluation and optimization of the printing parameters, the hydrogel was supplemented with bromophenol blue (Carl Roth GmbH + Co. KG, Germany). Hydrogel for use with cells was sterilized with a syringe filter (0.45 μm, Sartorius Stedim Biotech, Germany). 5% alginate/5% gelatin hydrogel was produced as described in Ouyang et al. (2016) and supplemented. In brief, gelatin (Sigma Aldrich, USA) was dissolved in pre-warmed (37°C) at a concentration of 10% (w/v). 1 ml of this solution were transferred into a 2 ml reaction tube (Eppendorf, Germany), covered with 100 mg alginate powder and supplemented with 0.3 ml PBS. This mixture was repeatedly heated up to 60°C and cooled down to 4°C until gelatin was completely dissolved and the gel exhibited a homogeneous consistency. The mixture was subsequently supplemented with HEK293^YFP^ cells as described below.

### Preparation of Cell-Laden Hydrogel

For biofabrication experiments, HEK293^YFP^ cells, previously dislodged from a T75 flask (TPP, Switzerland) using 0.25% trypsin–EDTA solution (Gibco BRL) and re-suspended into culture medium, were counted using a haemocytometer (LO Laboroptik GmbH, Germany).

For preparation of cell-laden 5% alginate hydrogel, the cell number was adjusted to 10^6^ cells/ml. In a next step, the cells were centrifuged for 5 min at 15 g, the supernatant was discarded using a vacuum pump and 1 ml 5% alginate hydrogel, prepared as described above, was added to the cells and was gently mixed using a 1 ml pipet.

For preparation of cell-laden 5% alginate/5% gelatin hydrogel, 1 ml pre-warmed (37°C) PBS was supplemented with 2.86^*^10^6^ HEK293^YFP^ cells and was used for mixing with 5% alginate and gelatine as described above.

### Preparation for 3D Printing

Prior to 3D printing, a coverslip (24 × 40 mm, Menzel GmbH, Germany) was attached to the printing bed of the device using sticky tape and was thoroughly cleaned with 70% ethanol. In a next step, a sterile and disposable 1-ml syringe (Injekt^®^-F, Braun) was filled with HEK293^YFP^ hydrogel and was mounted into the syringe unit. Subsequently, a sterile and blunt needle (Braun Sterican^®^ 0.4 × 25 mm or 0.8 × 40 mm) was attached to the syringe, and the distance between the needle and the printing bed was adjusted to leave a gap of approximately the height of a sheet of paper.

Bioconstructs were designed using CAD software (Autodesk Inventor Professional 2017, Autodesk, Inc., USA) and were translated into gcode using the open source software Cura 14.07 (Ultimaker, USA). The same software was used for parametrization of the printing process and generated gcode was transferred to the 3D printer using a common mini SD card, provided with the printer.

### 3D Printing

Cell-free and cell-laden hydrogels were printed at room temperature and at a speed of 550–600 or 3 steps/mm as well as an extrusion rate of 100 or 300 mm/s or onto the pre-attached and cleaned coverslip. Cell-laden hydrogels were printed under sterile conditions inside a standard laminar flow hood. An image of the ultra-low-cost 3D bioprinter during operation inside a laminar flow hood is depicted in Figure 3C. Immediately after printing, the coverslip was transferred into a 6 cm culture dish (TPP, Switzerland) and hydrogels were crosslinked by exposure to 0.1 M CaCl_2_ solution (Fluka Analytical, Germany). CaCl_2_ solution was removed after 20 min followed by a washing step in culture medium. 3D printed bioconstructs were cultured at 37°C, 5% CO_2_ in a humidified incubator.

### Microscopic Evaluation of 3D Printed Bioconstructs

Prior to microscopic evaluation, the culture medium was replaced by 3 ml Ringer's solution, which contained (in mM) NaCl 140, KCl 5, CaCl_2_ 2, MgCl_2_ 1, HEPES 10, and glucose 10 (pH 7.4, NaOH) and was exchanged by culture medium upon imaging.

For fluo-micrography, the 6 cm dishes were transferred to an imaging system (Nikon Eclipse Ti, Nikon, Japan), and contructs were imaged at room temperature and at 400 (20 × 20) overlapping locations with a 10x objective (CFI Plan Fluor DL 10X Phase, N.A. 0.30, Nikon, Japan) using the *large image* function of NIS Elements software (AR 4.00.08, 64-bit, Nikon, Japan). Constructs were scanned through acquisition of multiple tile images as the field of view of the employed objective is smaller than the size of the printed structures. Illumination from a xenon lamp (Lambda LS, Sutter Instruments, USA), passing through a filter block (C-FL Epi-FL FITC, EX 465-495, DM 505, BA 515-555, Olympus, Japan) was used to excite and detect YFP fluorescence signal. Fluorescence was recorded by a sCMOS camera (NEO, Andor, Ireland) and digitized to disk onto a standard personal computer (Dell Precision T3500, Dell, USA) with Windows 7 operating System (Microsoft Corporation, USA).

For transmission light microscopy, the culture dishes were placed onto the stage of a Nikon Eclipse TS 100 microscope (Nikon, Japan), and printed structures were imaged using a 4x objective and digitized to an SD card using a digital camera (EOS M10, Canon, Japan) attached to the microscope.

### Image Analysis

Images generated from microscopic evaluation were processed and quantitatively analyzed using FIJI 1.49s (ImageJ) software (National Institutes of Health, US).

Tiled images from fluorescence microscopy were processed as follows. In a first step, images were stitched using the ImageJ plugin *Grid/Collection stitching* (Preibisch et al., 2009). In a next step, the resulting reconstructed images were spatially aligned with ImageJ using the plugin *Register Virtual Stack*. For quantitative assessment of cellular proliferation, YFP fluorescence intensity in the stitched and registered images was analyzed within a line ROI (region of interest) using the *Plot Z-axis Profile* function of ImageJ and is expressed a relative fluorescence unit (RFU).

For assessment of the accuracy of our ultra-low-cost 3D bioprinter for printing 5% alginate hydrogel, vertically aligned structures visualized in transmission light images were manually selected as well as defined as a ROI and the area of selected ROIs was quantified using the function *Measure* in ImageJ. The average width (in pixels) of the selected ROI was calculated with the following equation:

(1) $$\begin{array}{r} width\;\left(struct.\right)=\;\frac{Area\;\left(struct.\right)}{length\;\left(struct.\right)} \end{array}$$

where *Area* is the area (in pixels) of the selected ROI and *length* is the height (in pixels) of the acquired image. Pixels values were subsequently converted into μm based on the pre-evaluated size of the pixels.

The accuracy of our system for printing 5% alginate/5% gelatin hydrogel was evaluated using vertically aligned structures visualized in transmission light images which were measured manually at 20 positions each and were subsequently translated into physical size using ImageJ software.

## Discussion

To overcome the limitations of commercially available technology suitable for 3D bioprinting we have developed a 3D bioprinter that is advantageous for several reasons. First, with a footprint of 400 × 415 mm and a weight of 8.5 kilogram, the system is smaller and lighter compared to conventional technology, e.g., the BioScaffolder 2.1 (GeSIM, Germany) and is thus, suitable to operation within a sterile environment, such as a standard laminar flow hood. Also, due to the aforementioned characteristics, the system is portable and suited, e.g., for shared-operation in different laboratories. Second, with ~€ 150 costs, our system is ultra-low-cost and is readily applicable to a broad range of laboratories in various fields of research and also educational institutions. Third, the device was built based on a *Makers* approach, i.e., by using off-the-shelf components and 3D desktop printing, and could easily be re-built or modified by individuals, for example in so-called fab labs (fabrication laboratories). Fab labs are small-scale workshops—also increasingly being adopted by schools as platforms for project-based, hands-on STEM (science, technology, engineering and mathematics) education—and provide support and infrastructure, e.g., for customization and CAD modeling. Fourth, the employed concept of “auto-generation,” i.e., using an off-the–shelf 3D printer for fabrication of parts to be used for modification and generation of a derived system with a different function, may also be applicable to other types of 3D printers. Also, as the device can easily be converted back into its original configuration, it can be used for manufacturing of replacement parts, i.e., for “auto-servicing,” as well as for further modification and customization. This novelty is also a reason for the printer probably being the most resource-efficient 3D bioprinter reported in the literature. As the original printing system accounts for >95% of the overall costs, future systems that are based on our concept may be even cheaper because the costs for desktop 3D printing systems are currently decreasing while their overall availability increases. Fifth, the printer in its current configuration is easy to use, straightforward, and does not require highly skilled staff for application and maintenance, further highlighting the applicability in the academic as well as educational field.

The ultra-low-cost printer is equipped with a disposable 1-ml syringe that is cost-effective and thus, supports the low-cost concept of the overall system. Also, the luer lock connector of the syringe allows operation with different needle types and supports experimental versatility. However, due to the fact, that the syringes and needles are not fabricated for use in high precision applications, both, syringe and needles vary in their lengths. Thus, the system in the current configuration requires calibration of the distance between needle and printing bed every time the syringe and/or needle is replaced, resulting is a preparatory lead time of several minutes upon mounting the syringe into the unit.

The device in its current configuration has been transported between institutes and used on a daily basis in different labs by differently skilled and experienced people, including graduate and undergraduate students, for several months without failure or the need for repair. This indicates that the system is robust and suitable to be used in schools and also academic research environments, e.g., in hands-on practical student courses.

Despite the above mentioned advantages over conventional devices, the presented system has also potential for improvement. When assessing the printing characteristics of the ultra-low cost 3D bioprinter, we observed high repeatability with respect to highly viscous 5% alginate/5% gelatin hydrogel, but rather large deviation of the printed structures from the targeted dimensions that is probably due to the following reasons. First, the syringe unit is fabricated from flexible ABS and thus, prone to deformation during operation. Second, the threaded rod and connected nuts are of warehouse quality causing the components to slip and to produce varying pressure applied to the hydrogel. Also, the spring coupling used to connect the threaded rod with the stepper motor contributes to inconsistent results through unpredictable mechanical coupling compliance. Third, syringe and plunger are both made of plastic that it is prone to deformation during hydrogel extrusion, strongly affecting the pressure applied to the hydrogel and thus, the amount of extruded material. The aforementioned issues may be addressed by using a glass, e.g., Hamilton-syringe, and building the unit from aluminum and higher precision components including a threaded spindle with lead screw, terminally centered using ball bearings. It has been reported that the needle length affects cellular viability, with increasingly affected cellular viability along with increasing needle length (Faulkner-Jones et al., 2015). We used rather long needles of 24 mm length, hence, application of shorter needles may increase cell fitness and with that decrease variability of the device and printing results. Also, the shape of the needle may be modified to a conically shaped needle instead of the cylindrically shaped one we used, potentially reducing mechanical stress for extruded cells, increasing cellular viability (Reid et al., 2016) in printed constructs and with that the overall reliability of the system. Despite the fact that the aforementioned modifications would inevitably result in a 3D bioprinter of increased precision, it would also dramatically increase the costs and time required for its installation. Therefore, the presented system provides a smart balance between performance and costs. It is important to mention, that further testing with different materials is required for the modified printer to become more widely applicable. Consequently, this will also challenge the resolution issues encountered and mentioned above.

Our case studies with cell-laden hydrogels clearly indicate that both, construction of two- and three-dimensional objects is feasible using the ultra-low-cost system as well as that cell proliferation in long-term cultures follows an exponential function, indicating high cellular viability within generated bioconstructs. For two-dimensional constructs generated using 5% alginate bioink, the fluorescence signal increases by ~250% within 1 week (7 days *in vitro*). In contrast, the fluorescence signal originating from three-dimensional objects printed using a mixture of alginate and gelatin increases by >200% within a culture period being approximately three times longer (19 days *in vitro*) compared to the other constructs. The phenomenon of a much smaller proliferation rate within two- vs. three-dimensional constructs is somewhat expected for different reasons. First, the contact area of two-dimensional constructs with the surrounding culture medium, is larger compared to three-dimensional objects, increasing the availability of nutrients and promoting build-up of cellular metabolites potentially affecting cellular viability. Second, the viscosity of 5% alginate hydrogel is much lower compared to the alginate/gelatin mixture, also facilitating exchange of nutrient and metabolites. Third, a higher viscosity may affect morphogenesis during cell division as well as cell migration, affecting cellular proliferation, and resulting in an overall lower proliferation rate. Forth, the initial cell number or viability is strongly affected in three- compared to two-dimensional constructs potentially due to shear stress during printing as reflected by a reproducible decrease to ~70% of the original fluorescence signal in 2D vs. 3D objects (see Figures 3B, 4C). This phenomenon is somewhat expected as it has previously been reported by a number of studies (Irvine et al., 2015; Zavazava et al., 2015). However, it affects the number or viability of cells in the newly generated object and thus adds up to, i.e., multiplies, the aforementioned reasons, negatively influencing cellular proliferation rate.

The cell-laden bioconstructs fabricated in the course of this study are small compared to the dimensions of objects that may be fabricated based on the possible printing dimensions of the ultra-low-cost system as well as the volume of the implemented syringe. To demonstrate that the presented device in the used configuration is also suitable to printing of larger objects we generated a variety of differently sized and shaped constructs. Although these constructs only provide a small collection of examples, they highlight the applicability of the described technology and thus display its versatility and potential value or contribution to the emerging field of 3D-bioprinting.

Although all printed geometries—including mesh-structures, triangle, rectangle, pentagon, circle, cylinders, and even an upside-down pyramid with arching overhangs—are clearly apparent and discernible, the surface is of the depicted objects is rough and not always regular. This is due to the employed hydrogel and thus, independent of the used 3D-bioprinter. This phenomenon has also be reported in the literature for other bioprinting devices and fabricated 3D objects (see e.g., Duan et al., 2013; Mannoor et al., 2013; Itoh et al., 2015; Kim et al., 2018).

## Conclusions

Here, we describe the conversion of an off-the shelf 3D printer into a 3D bioprinter, providing a starting point for an exchangeable concept for further modification and optimization by the community to fit the specific requirements of an individual question in biomedical research. This is the first time that a 3D bioprinting system is described, which is ultra-low-cost and thus available to a broad community, largely “auto-generating” and “auto-servicing,” hence resource-efficient, portable for e.g., shared use and customizable through provided design (STEP) and print (STL) files. We applied the technology with recombinant human embryonic kidney-derived cells stably expressing YFP embedded into different alginate-based hydrogels. Qualitative and quantitative analysis of transmission images and fluo-micrographs generated from microscopic evaluation of newly fabricated and long-term cultured bioconstructs clearly demonstrated its applicability with the employed cell line, hydrogels and within the specified range of model dimensions. However, upon further testing the presented technology could also be used with other cell types and bioinks and could also be extended by implementation of further instrumentation including devices for calibration of the needle-printing bed distance or temperature control infrastructure, for cooling or heating of the employed bioink. As the syringe unit was constructed based on a *Makers* approach and all CAD files are provided with this paper, also other biosensor technology to suit the requirements for a vast variety of fields of research including life and even material sciences, could be implemented.

In summary, the ultra-low-cost 3D bioprinting platform presented in this article improves the classical technologies in terms of portability, cost and customizability and provides an example of low-cost biofabrication technology that is compatible with fast and resource-efficient prototype optimization. Altogether, this work contributes to expanding the applicability and availability of commercially viable 3D bioprinting devices for use in biomedical research and/or education.

## Data Availability

The datasets generated for this study are available on request to the corresponding author.

## Consent for Publication

Written informed consent was obtained from the individual depicted in Figure 3C for the publication of the image.

## Author Contributions

DG conceived the original idea and supervised the project, assembled the system, and wrote the paper. PH created 3D CAD models. OF provided materials and contributed to the final version of the manuscript. MK and MG conducted experiments. MK, MG, and DG analyzed and displayed the data. All authors agreed on the final version of the manuscript.

### Conflict of Interest Statement

The authors declare that the research was conducted in the absence of any commercial or financial relationships that could be construed as a potential conflict of interest.
