# Supplementary material for: Ultra-Low-Cost 3D Bioprinting: Modification and Application of an Off-the-Shelf Desktop 3D-Printer for Biofabrication
Source: Front Bioeng Biotechnol. 2019 Jul 31;7:184. doi: 10.3389/fbioe.2019.00184 (PMC6684753; doi:10.3389/fbioe.2019.00184)
Supplement: Supplementary file 2 [file Data_Sheet_2.pdf]

Ultra-low-cost 3D bioprinting: Modification & application of an off-the-shelf desktop 3D-printer for biofabrication

Melanie Kahl[a+], Phillip Hoyer[a+], Oliver Friedrich[a] and Daniel F. Gilbert*[a]

[a] *Institute of Medical Biotechnology, Friedrich-Alexander-University Erlangen-Nürnberg, Erlangen, Germany.*
** Correspondence to: Dr. Daniel Gilbert, Institute of Medical Biotechnology, FAU Erlangen-Nuremberg, Erlangen, Germany, daniel.gilbert@fau.de*
[+] *contributed equally*

Supplementary Information

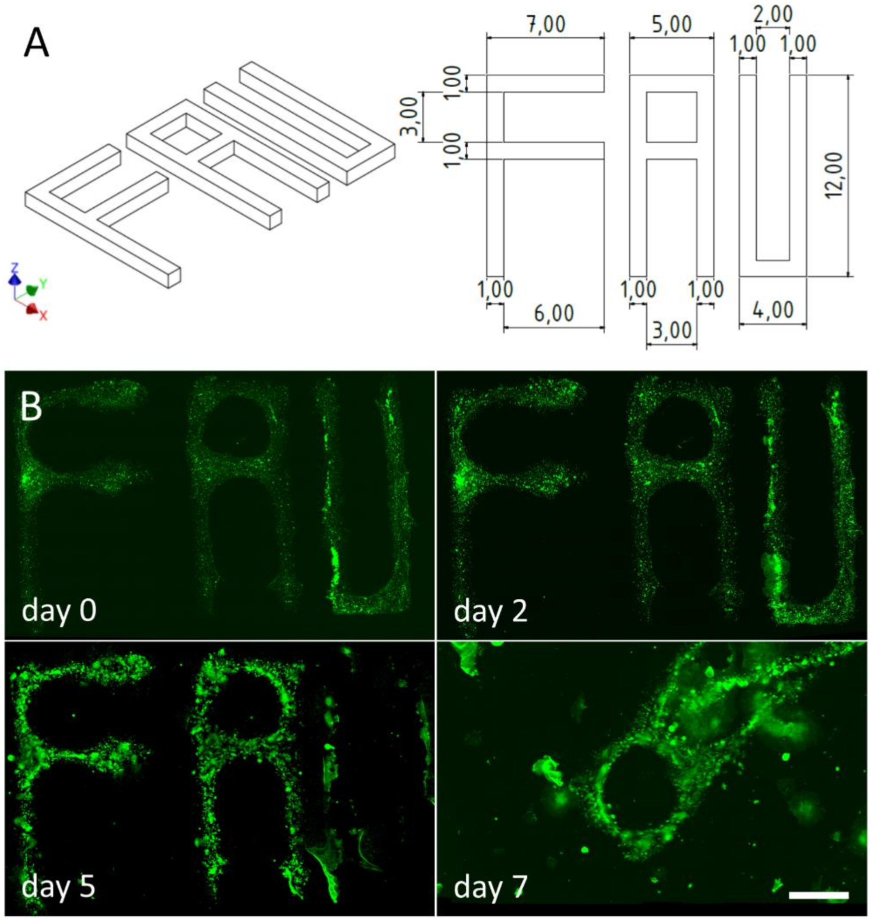

Figure S1. Maturation of 3D printed recombinant HEK293^{YFP} cell-laden alginate-hydrogel. A. Three-dimensional representation (left) and dimensions (right) of a bio-construct shown in (B) and printed with our ultra-low-cost 3D-bioprinter. The letters FAU abbreviate Friedrich-Alexander-University Erlangen-Nuremberg. **B.** Reconstructed fluo-micrographs of the printed structure shown in (A) after 0, 2, 5 and 7 days *in vitro* maturation, respectively, indicating cellular proliferation and viability within the bio-construct. These data further demonstrate the suitability of the in house-modified system for biomedical research. The large structure dislodges, disintegrates and floats in the culture medium after approx. 5 days in culture due to weak attachment to the glass coverslip, followed by. Scale bar: 3 mm.

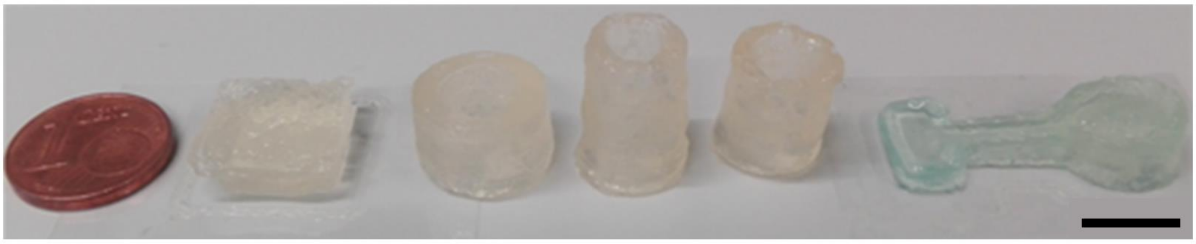

Figure S2. Examples of printed objects further highlighting the applicability of the in house-modified system for fabrication of 3D constructs. From left to right: one euro cent for comparison; half-cut and upside-down pyramid; cylinders of different diameters and heights; spoon-shaped objects. Scale bar: 10 mm.

Table S1. Detailed parts list

Part	File name / DIN	No.	Dimensions	Material [g]	Printing time [hour:min]
Connector	Slider for plunger	1		27.64	2:33
	Hold carriage to carriage	1		5.06	0:30
	Motor connector adapter	1		15.01	1:28
	Motor connector	1		9.99	1:02
	Screws DIN EN ISO 4762	4	M3x60		
	Screws switch DIN 912	2	M2x10		
Carriage	Slider BOTTOM	1		1.67	0:08
	Slider LARGE	1		2.52	0:16
	Slider SMALL	1		2.87	0:18
	Square nut DIN 557	2	M5		
	Screw DIN EN 24017	4	M3x20		
	Nut DIN EN 24036	4	M3		
Plunger connector	Plunger connector TOP	1		2.67	0:17
	Plunger connector BOTTOM	1		1.53	0:11
	Plunger connector LID	1		0.31	0:04
	Screw DIN EN ISO 4762	2	M3x16		
	Nut DIN EN 24036	3	M3		
	Thumb screw DIN 653	1	M3x16		
Syringe carrier	Syringe carrier BOTTOM	1		2.00	0:29
	Syringe carrier MIDDLE	1		3.99	0:20
	Syringe carrier TOP	1		3.51	0:17
	Syringe carrier LEVER	2		0.21	0:02
	Screw DIN EN ISO 4762	2	M3x25		
	Nut DIN EN 24036	2	M3		
Misc	Flexible Helical Coupling	1	M5/M8		
	Stepper motor	1			
	Threaded rod	1	M5x125		
	Screw printer connector ISO 7380	4	M5x6		
